# Crystal Structure of the ATPase Domain of the Human AAA+ Protein Paraplegin/SPG7

**DOI:** 10.1371/journal.pone.0006975

**Published:** 2009-10-20

**Authors:** Tobias Karlberg, Susanne van den Berg, Martin Hammarström, Johanna Sagemark, Ida Johansson, Lovisa Holmberg-Schiavone, Herwig Schüler

**Affiliations:** Structural Genomics Consortium, Department of Medical Biochemistry and Biophysics, Karolinska Institutet, Stockholm, Sweden; University of Cambridge, United Kingdom

## Abstract

Paraplegin is an *m*-AAA protease of the mitochondrial inner membrane that is linked to hereditary spastic paraplegias. The gene encodes an FtsH-homology protease domain in tandem with an AAA+ homology ATPase domain. The protein is believed to form a hexamer that uses ATPase-driven conformational changes in its AAA-domain to deliver substrate peptides to its protease domain. We present the crystal structure of the AAA-domain of human paraplegin bound to ADP at 2.2 Å. This enables assignment of the roles of specific side chains within the catalytic cycle, and provides the structural basis for understanding the mechanism of disease mutations.

**Enhanced version:**

**This article can also be viewed as an enhanced version in which the text of the article is integrated with interactive 3D representations and animated transitions. Please note that a web plugin is required to access this enhanced functionality. Instructions for the installation and use of the web plugin are available in Text S1.**

## Introduction

Molecular machines are protein assemblies, often multimeric complexes, that convert chemical energy into mechanical work by undergoing conformational changes in their protein subunits. An ATPase domain, alone or coupled in series, usually forms the basis for these machineries. The AAA+ domain (‘ATPases associated with diverse cellular activities’ and related proteins) is one such module; it is often found as a hexameric ring complex, and couples ATP hydrolysis to activities that involve protein remodeling (reviewed in [1]–[4]). Generally, AAA+ ATPases bind their native targets, unfold them locally by ATP hydrolysis-driven conformational changes, and release the unfolded target for further processing (such as refolding, transport across membranes, or degradation). AAA+ domains are found in all kingdoms of life; in eukaryotes, they are present in the cytosol, in most cellular compartments, as well as associated with membranes. Over 60 classical AAA+ members and an additional number of divergent domains are encoded in the human genome.

**Figure 1:**
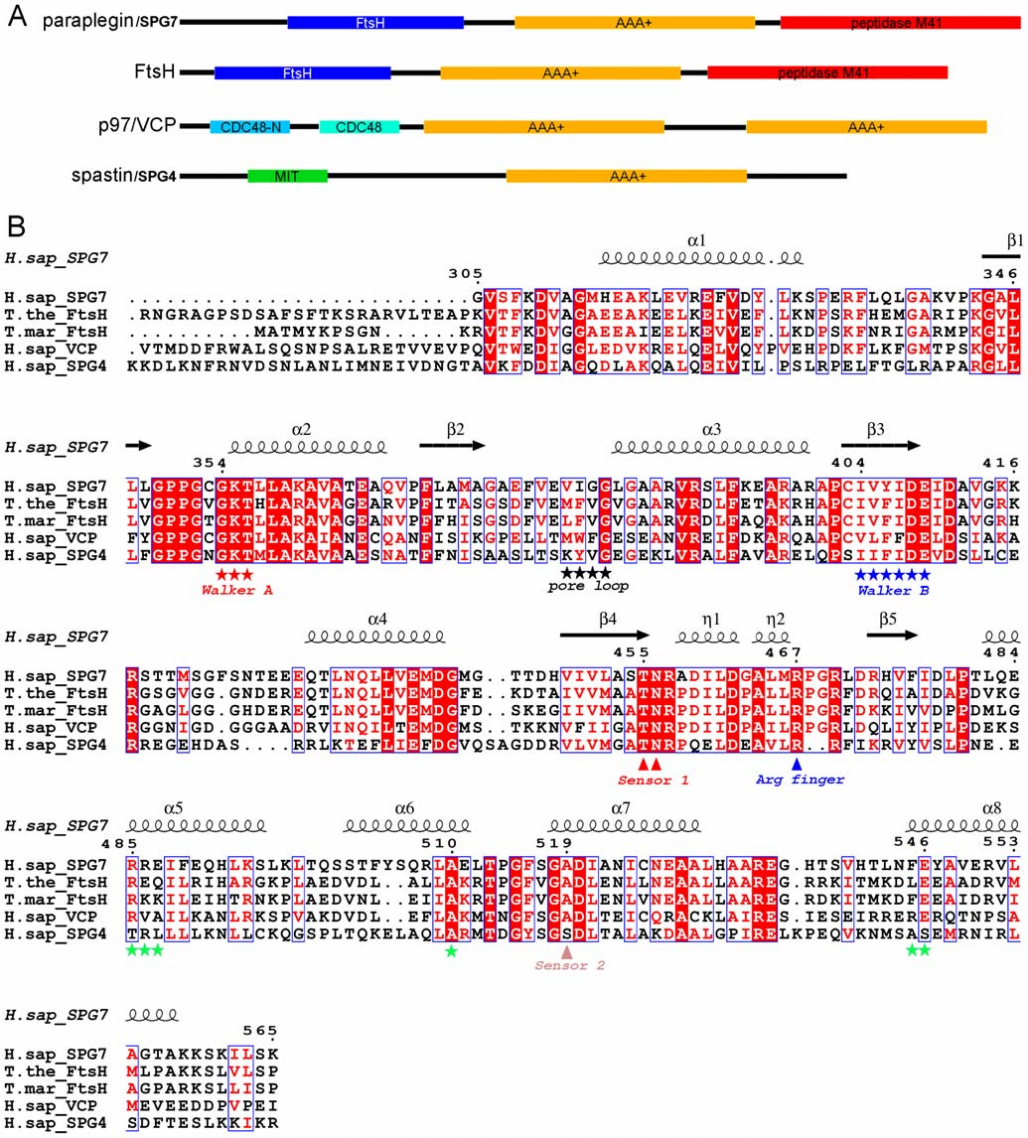
Domain arrangement and sequence comparison of paraplegin/SPG7 and related AAA+ proteins. A. Domain arrangement of paraplegin, FtsH, VCP, and spastin. Homologies included are the FtsH extracellular (Pfam entry PF36480), AAA+ (PF00004), metallopeptidase M41 (PF01434), Cell division protein-48 (CDC48) N-terminal (PF02359), CDC48-2 (PF02933), and microtubule interacting and transport (MIT; PF04212) domains. B. Sequence alignment of the ATPase domains of paraplegin and related proteins to illustrate the positions of conserved residues. Residue numbering and secondary structural elements are indicated for paraplegin (PDB entry 2qz4) above the alignment. Walker A and B, and Sensor 1 and 2 motifs, the arginine residue predicted to act as an arginine finger, as well as the pore loop are indicated below the alignment. Indicated by green asterisks are HSP disease related positions. Sequences shown are human paraplegin/SPG7 (residues 305–565; PDB entry 2qz4; gene identification code 116242796). *Thermus thermophilus* FtsH (126–624; 2dhr; gi:8051696), *Thermotoga maritima* FtsH (147–610; 2cea; gi:15643346), human p97/VCP (116–417; gi:112818458), and human spastin/SPG4 (114–437; gi:11875211).

Human paraplegin (SPG7) [5] is a mitochondrial membrane-associated AAA-domain containing metalloprotease consisting of three homology domains (Figure 1): The N-terminal FtsH-extracellular domain, which is found in membrane-bound ATP-dependent proteases; the intermediate AAA-domain; and the C-terminal metallopeptidase M41 domain [6]. The current state of evidence indicates that the protein forms cylindrical hexamers that insert in the inner mitochondrial membrane such that the FtsH-domains are located in the lumen, and the catalytic domains in the matrix. Paraplegin is implicated in the degradation of proteins that come out misfolded after transport across the mitochondrial membranes, and in cleavage of mitochondrial targeting sequences. Paraplegin function is critically involved in ribosome maturation [7].

Paraplegin was originally discovered and named for its involvement in hereditary spastic paraplegias (HSP) [5]. These are heterogeneous syndromes most commonly manifested in progressive spasticity and weakness of the lower limbs. At present, around 40 genes have been recognized to contribute to HSP [8]. HSP-related mutations in the SPG7 gene cause axonal degeneration (reviewed in [9]). Nonsense loss-of-function mutations are prevalent, but disease-linked amino acid replacements in the AAA-domain have also been identified.

Here, the crystal structure of the AAA-domain of human paraplegin is presented in complex with ADP. The overall fold and the nucleotide binding site are described. We outline the side chains that, by homology with FtsH, are implicated in hexamer formation, substrate binding, and chemomechanical coupling. Finally, we discuss the putative roles of disease-related residues.

## Materials and Methods

### Protein expression and purification

Human SPG7 cDNA was obtained from Deutsches Ressourcenzentrum für Genomforschung (accession no. BC036104). The sequence encoding paraplegin residues 305–565 was amplified by PCR and inserted into pNIC28-Bsa4 by ligation independent cloning. The expression construct included a TEV protease-cleavable N-terminal hexahistidine tag. Protein expression in *Escherichia coli* strain BL21(DE3) gold pRARE2 was done in a LEX system (Harbinger Biotechnology and Engineering) using Terrific Broth medium supplemented with 8 g/l glycerol, 34 µg/ml chloramphenicol and 50 µg/ml kanamycin, induction with 0.5 mM IPTG, and over night culture at 18°C. Cell pellets were resuspended in 50 mM HEPES pH 7.8, 500 mM NaCl, 10 mM imidazole, 10% glycerol, 0.5 mM TCEP, and Complete EDTA-free Protease Inhibitor (Roche Biosciences). Cells were lysed by a freeze/thaw cycle followed by addition of benzonase (Novagen) and sonication (Sonics VibraCell). Lysates were centrifuged at 49,000 g for 20 min at 4°C. The supernatants were decanted and filtered.

**Figure 2:**
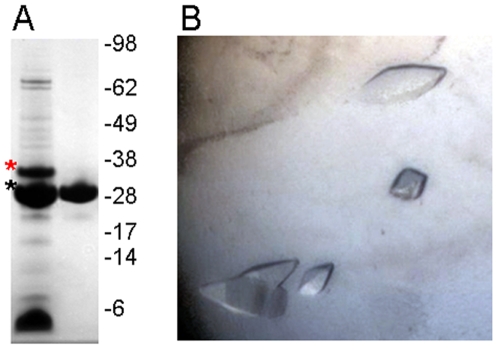
Purification and crystallization of paraplegin^305–565^. A. Coomassie-stained SDS-polyacrylamide gel showing the purity of crude paraplegin^305–565^ after TEV-cleavage (left lane; red asterisk, hexahistidine-tagged protein; black asterisk, cleaved protein), and after the final purification step (right lane). B. Example of crystals grown under the conditions that yielded diffraction data.

Filtered lysates of cells expressing paraplegin^305–565^ were loaded onto HiTrap Chelating HP columns (GE Healthcare) in buffer 1 (30 mM HEPES pH 7.5, 500 mM NaCl, 10 mM imidazole, 10% glycerol, and 0.5 mM TCEP). The columns were washed with buffer 1 containing 25 mM imidazole. Bound protein was eluted with buffer 1 containing 500 mM imidazole. The protein showed a strong tendency to precipitate in the presence of imidazole. The sample was filtered and loaded onto a HiLoad 16/60 Superdex-200 column (GE Healthcare) pre-equilibrated with buffer 2 (50 mM sodium citrate pH 5.5, 100 mM ammonium sulfate, 10% glycerol, 2 mM TCEP). Fractions containing target protein were pooled, and the N-terminal hexahistidine tag was removed by incubation with His-tagged TEV protease (molar ratio 50∶1) over night at room temperature, followed by passage over a 1 ml HisTrap HP column in buffer 1 without imidazole (Figure 2A). Purified protein was concentrated using Vivaspin (Sartorius) centrifugal concentrators. Aliquots were flash-frozen and stored at −80°C. All proteins were verified by time-of-flight mass spectrometry analysis.

### Crystallization, data collection, structure solution and refinement

Crystals of paraplegin^305–565^ in complex with ADP were obtained by vapor diffusion in sitting drops incubated at 20°C by mixing equal amounts of protein solution at (20.0 mg/ml, including 2.5 mM ATP and 2.5 mM MgCl_2_) and reservoir solution containing 25% PEG-3350, 100 mM Bis-Tris pH 6.5, 200 mM ammonium acetate (Figure 2B). Crystals appeared after three days and continued to grow for one more week to reach their maximal size (0.15 mm×0.06 mm×0.06 mm). Reservoir solution supplemented with 15% glycerol was added directly to the drop, and crystals were mounted and flash-frozen in liquid nitrogen. Diffraction data to 2.2 Å resolution were collected at ESRF Grenoble, France (beamline ID 14-2). Data were integrated and scaled using XDS [10]; one molecule was found in the asymmetric unit. The structure was solved by Molrep [11] using the structure of *T. maritima* FtsH (PDB entry 2ce7) as a search model. The structure was refined with RefMac5 [12]. Restrained refinement using three TLS groups was performed using the TLSMD server [13]. Model building was done using Coot [14]. For further details, see Table 1. Geometry of the models was analyzed with Molprobity [15]. Sequence alignments were obtained using ESPript [16].

**Table 1:** Paraplegin crystal structure: Data collection and refinement statistics.

Structure	Paraplegin
Ligand	ADP
PDB entry	2QZ4
Beamline	ID 14-2
Wavelength (Å)	0.9330
Space group	P4_3_22
Cell dimensions	
*a*, *b*, *c* (Å)	57.2, 57.2, 157.2
α, β, γ (°)	90.0, 90.0, 90.0
Resolution (Å)	40.0–2.2 (2.3–2.2)
R_sym_	0.069 (0.203)
I/(σI)	23.7 (7.2)
Completeness (%)	99.1 (62.7)
Redundancy	10.6 (4.7)
Refinement	
Resolution (Å)	39.1 – 2.2
No. reflections	12529
R_work_ †/R_free_ ‡	0.209/0.269
No. atoms	
Protein	1680
Ligands	27
Water	55
*B*-factors (Å^2^)	
Protein	56.1
Ligands	40.2
Water	55.6
R.m.s. deviations	
Bond lengths (Å)	0.015
Bond angles (°)	1.6
Ramachandran plot	
Favored regions (%)	94.9
Disallowed regions (%)	0.9

Values for the highest resolution shell are shown in parentheses.

† R_work_ is defined as Σ||F_obs_|−|F_calc_||/Σ |F_obs_|, where F_obs_ and F_calc_ are observed and calculated structure-factor amplitudes, respectively.

‡ R_free_ is the R factor for the test set (5–10% of the data).

For further details, please refer to http://www.thesgc.com/SGC-WebPages/StructureDescription/MM-v2.php?pdb=2QZ4.

### Data deposition

The atomic coordinates and structure factors have been deposited with the Protein Data Bank, www.rcsb.org (PDB entry code 2qz4).

## Results and Discussion

**Figure 3:**
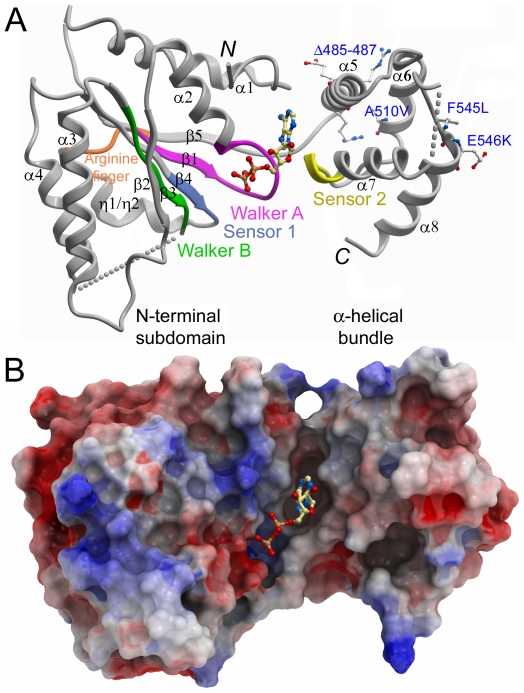
Overview of the paraplegin ATPase domain structure. A. Schematic representation of the crystal structure of a monomer of paraplegin^305–565^ with bound ADP. Sequence motifs indicated in the sequence alignment in Figure 1 have been mapped onto the structure. The positions of disease-related residues are labeled in blue. B. Electrostatic surface representation of paraplegin^305–565^ illustrating the nucleotide binding cleft.

We purified recombinant human paraplegin including residues 305–565 (corresponding to the ATPase domain; Figure 1A) in the absence of excess nucleotide. Elution profiles after gel filtration suggested the presence of a minor amount of higher molecular weight complex, while the majority of the protein eluted as a monomer. We determined the crystal structure of this protein to a resolution of 2.2 Å. The protein crystallized with one monomer in the asymmetric unit, and ADP was bound in the nucleotide binding site. The structure shows the typical AAA+ domain consisting of a five-stranded parallel β-sheet flanked by two α-helices on each side, together with a carboxy-terminal bundle of four antiparallel α-helices (Figure 3 and Supplementary Datapack S1). The refined structure contains residues 305–565, with the exception of loop residues Arg 333 – Gly 338, Asp 411 – Glu 429, Ala 532 – Ser 539, and Ser 560 – Lys 565, for which no density could be observed.

### Nucleotide binding site and the conserved motifs

**Figure 4:**
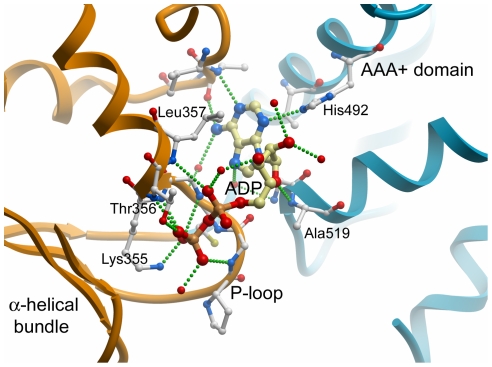
The nucleotide binding site of paraplegin. Details of side chain interactions with ADP.

The nucleotide binding pocket is located at the interface between the β-sheet and the α-helical bundle (Figure 3). Despite high concentrations of ATP and Mg^2+^ during crystallization, ADP was clearly seen in the electron density, and as in previously determined FtsH structures [17], density for a magnesium ion was not observed. ADP interacts with side chains from both the amino-terminal part of the AAA-domain and the carboxy-terminal α-helical bundle (Figure 4 and Supplementary Datapack S1).

The conserved motifs of AAA+ domains are located in the proximity of the nucleotide binding cleft (Figure 3). The conserved P-loop in the Walker A motif (GxxGxGK(T/S); residues Gly 350 – Thr 356), is central in binding the β–phosphate of ADP through hydrogen bonding interactions with the backbone nitrogens of residues 352 – 356, the Lys 355 and Thr 356 side chains, and a water molecule (Figure 4). The α-phosphate binds to the backbone nitrogen of Leu 357 and a water molecule. The two hydroxyls of the ribose moiety hydrogen bond with three water molecules and the ribose 4′-oxygen hydrogen bonds with the Ala 519 backbone amide. The adenine base is stacked between the hydrophobic side chains of Leu 357 and Ile 448, and hydrogen bonds with the His 492 side chain, the backbone amide and oxygen of Ala 312, and two water molecules.

The side chains of Lys 355 and Thr 356 in Walker A motif make particularly important contributions to β-phosphate binding (Figure 4). Mutational analysis of the p97/VCP lysine residue corresponding to paraplegin Lys 355 shows that the side chain is essential for ATP hydrolysis [18]. Interestingly, in a subset of AAA+ proteins, the Ser/Thr residue of the P-loop is replaced by an asparagine.

The Walker B motif Ile 404 – Glu 409 (IVYIDE) located to β-strand 3 is highly conserved among AAA+ proteins. The acidic side chains in this motif generally coordinate a magnesium ion that activates a water molecule for nucleophilic attack on the ATP γ-phosphate.

Sequence Ser 454 – Asn 456 corresponds to the AAA+ Sensor 1 motif (STN). In FtsH, the asparagine is situated at the interface between the ATPase domain and the protease domain, interacting with the γ-phosphate of ADPNP [19], [20]. This sensor asparagine is a predicted key residue in ATP hydrolysis [21]. The corresponding Asn 456 side chain of paraplegin contacts the β-phosphate by a water-mediated interaction.

The Sensor 2 motif Gly 518 - Asp 520 (GAD) couples the state of nucleotide hydrolysis to the oligomerization state. Sequence alignments suggest that the sensor 2 arginine is mutually exclusive with the second Arg near the arginine finger (Arg 467 – Arg 470; RPGR). Thus AAA+ domains contain either one Arg at the C-terminus of α5 and the sequence GARx at the N-terminal end of α8 (as in e.g. HslU, RuvB and DnaA), or the sequences RxxR and xGAD/E (as in e.g. paraplegin, FtsH and p97) [22].

### Role of the alpha-helical bundle

The carboxy-terminal α-helical bundle domain is present in essentially all known AAA+ ATPases [23]. Together, the helical bundle and the larger N-terminal section of the ATPase domain enclose the nucleotide binding cleft (Figure 4). Comparison of different AAA+ domain structures shows that the spatial arrangement of the α–helical bundle relative to the N-terminal section of the ATPase domain can vary considerably. Superposition of the N-terminal section of the paraplegin ATPase domain with the corresponding domain of *Thermus thermophilus* FtsH (1iy1) reveals a difference of up to 35 degrees between individual corresponding helices of the α-helical bundle. This reflects the mobility of the linker between these domains and shows their potential role in closure of the ATP binding cleft. Although there is no correlation between cleft closure and the state of ATP hydrolysis in the AAA+ domain structures solved to date, the binding of ATP and release of ADP most likely have an effect on the spatial arrangement of the bundle, which in turn could affect the protease domain.

### Closest structural homologues

A structural homology search using the Dali server [24] reveals that, overall, our structure is most similar to structures of bacterial FtsH (*Thermotoga maritima*, 2cea, 2ce7; *Helicobacter pylori*, 2r65; *T. thermophilus*, 2dhr, 1iy0; *Escherichia coli*, 1lv7), p97/VCP (in particular the D2 subunit; 3cf0), human fidgetin-like protein-1 (3d8b) and the human VPS4/SKD1 ATPase (1xwi). Pairwise superposition of the Cα-positions within the AAA+ domains of these structures gives rms differences between 1.7 and 2.8 Å. The structures of the closest paraplegin homologs are those of bacterial FtsH [17], [25], which are 53% identical and >70% similar on the protein sequence level.

FtsH, as paraplegin, forms hexamers. The FtsH AAA-domain has previously been determined as either a monomer (1lv7, 2dhr) or a monomer arranged along a 6_5_-screw axis [17], while the AAA-domain and the protease domain in tandem crystallized as a hexameric double donut, with the AAA-domain ring overlying the protease ring [20], [25]. The upper AAA-ring is not flat in either of these structures; rather, the monomers are staggered in either 2-fold or 3-fold symmetry, which has been proposed to reflect post-hydrolysis conformations [20], [25]. Collectively, these results show that the protease domain makes a large contribution to intermonomer contacts, and suggest that the ATPase domains have a degree of flexibility also within the context of the ring.

### Intermonomer contacts and progressive ATP hydrolysis

**Figure 5:**
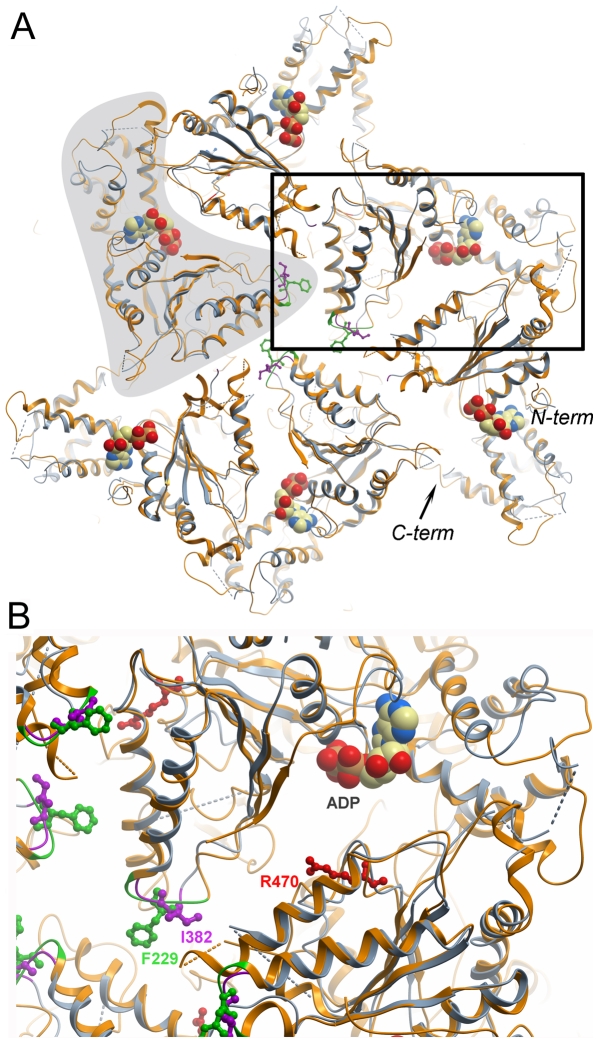
Model of the paraplegin hexamer. A. The hexameric structure of paraplegin^305–565^ was modelled by aligning our crystal structure (blue) with each monomer within the *T. thermophilus* FtsH hexamer crystal structure (2dhr; orange). The outline of one monomer is indicated by grey shading, and the N- and C-termini of another monomer are indicated. The boxed area is expanded in panel B. B. Close-up of the region around the pore loops and the monomer interface around the nucleotide binding site. The hydrophobic pore loop residue Phe228 of FtsH, implicated in substrate binding, is shown in green, and the corresponding paraplegin residue Ile832 is shown in purple. Paraplegin Arg470, shown in red, is a putative arginine finger that activates ATP hydrolysis in the neighbor monomer following a conformational change in the ring structure.

Given the high degree of similarity between paraplegin and FtsH, superposition of our paraplegin AAA-domain structure with the hexameric ring structures of FtsH (2dhr; 1iy1) can reveal common mechanism as well as distinct properties of paraplegin. This analysis shows that in the paraplegin hexamer, the N-terminus and the C-terminal α-helical bundle are expected at high radius, while the segment between β2 and α3 are predicted to lie near the central pore (Figure 5). The α-helical bundle has a threefold role during the chemo-mechanical cycle: 1. A conserved arginine at the N-terminal end of the bundle has been suggested to act as an arginine finger, extending into the active site of the neighboring subunit within the ring and stabilizing the charge developing during the transition state of ATP hydrolysis [19], [22]. In paraplegin, Arg 470 is positioned to act as an arginine finger. 2. As detailed above, sensor 2 motif (at the N-terminus of α7) is likely involved in coupling conformational changes resulting from ATP hydrolysis to the neighbor monomer within the AAA-ring. 3. The bundle is positioned to couple conformational changes resulting from ATP hydrolysis to the underlying protease ring.

### Substrate binding in the central pore of the hexamer

The residues at the N-terminus of α-helix 3 form the so-called pore loop of the ATPase domain. In the hexamer structure this pore mediates access to the catalytic residues of the peptidase domain [20]. Mutational analysis of FtsH and ClpX has shown that a bulky apolar residue at position 229 and a glycine at position 231 are essential for substrate-stimulated ATP hydrolysis as well as for substrate degradation [26], [27]. Inspection of the structure suggests that the bulky pore side chains and the nucleotide phosphate tail are mutually connected through the Walker B motif. The pore loop of the paraplegin AAA-domain contains the sequence Ile 382 – Gly 384 (IGG), and alignment of our structure with the FtsH hexamer show that the Ile 382 side chains of paraplegin line the central pore in a similar fashion as the Phe 229 side chains of FtsH (Figure 5). Most AAA+ proteins have aromatic side chains in this position, but paraplegin shares the isoleucine with the LON proteases and the D1 AAA-domain of p97. The residue at this position is an important determinant of substrate specificity [27].

### Roles of residues involved in hereditary spastic paraplegia

Most of the known disease mutations in the SPG7 gene lead to premature termination or map to the metallopeptidase domain. Some mutations, however, have been identified in the AAA+ domain, and surprisingly, they all map to the C-terminal α-helical bundle (Figure 3A, Figure 1B and Supplementary Datapack S1). The positions of these disease mutations will be discussed in the following paragraphs.

A three-residue deletion (Δ485–487) in the N-terminal end of α-helix 5 [28] would shorten the helix by one helical turn. In our structure, the arginine side chains make contacts with the adjacent helix-6 and the turn following it, while the glutamate carboxyl hydrogen bonds with the backbone amide of Gly 313 and with the His 315 side chain. These interactions are critical because they both stabilize the α-helical bundle and couple it to the N-terminus, and we predict that the Δ485 – 487 mutant leads to considerable destabilization of the overall structure of the AAA-domain.

The A510V mutation [28]–[30] maps to the C-terminal end of α-helix 6. The Ala 510 side chain lies on the buried surface of α6 that faces α5, and it is conceivable that a valine side chain in this position would disturb the hydrophobic core interactions in the helical bundle by its larger volume. In addition, it would possibly clash with the Arg 486 side chain discussed above.

The F545L [30] and E546K [31] mutations, at the N-terminal end of α-helix 8, are more elusive. Phe 545 is involved in the hydrophobic core of the α-helical bundle, but although the side chain does not form stacking interactions with other aromatic side chains in the vicinity, the Leu side chain in position 545 still causes the disease phenotype. A possible explanation for this is that, similar to the A510V mutation, the change in side chain volume might significantly destabilize the hydrophobic core of this minidomain. Glu 546 is situated on the outward facing surface of α8, and its role in disease might be explained by a possible disturbance of intermonomer contacts, by removal of the small acidic patch formed together with Glu 550, at some stage during the conformational cycle of the hexamer ring.

In summary, the disease mutations point to the important role of the C–terminal α-helical bundle of the AAA-domain, which is implicated in coupling the state of ATP hydrolysis to both the neighboring monomer within the ring, and the protease domain in the underlying ring. It is surprising that no disease mutations in the sites that are essential for ATP binding and hydrolysis have been identified. AAA+ proteins have ATPase activities that are orders of magnitude lower than other NTPases that feature the same conserved motifs. Thus possibly *in vivo*, ATPase-deficient paraplegin mutants are efficient enough to prevent the disease phenotype as long as the integrity of the pore loops and the conformational coupling between monomers and between the rings is maintained. Such mutants would be able to “ratchet” substrates through the pore and feed them to the peptidase with reduced ATPase activity, and thereby maintain the most important functions of paraplegin. However, disease mutations in the pore loops have not been identified to date. Paraplegin deficient mice develop progressive motor impairment, but are viable and live over 2 years [32]. Thus paraplegin function is at least partially redundant, and its loss might be in part compensated for by related proteins. Candidate proteins are the paraplegin-like protein AGF3L2, which is capable of hetero-oligomer formation with paraplegin [33], [34], and the presenilin-associated metalloprotease YME1L1.

## Supporting Information

Datapack S1 Standalone iSee datapack - contains the enhanced version of this article for use offline. This file can be opened using free software available for download at http://www.molsoft.com/icm_browser.html.
(ICB)

Text S1 Instructions for installation and use of the required web plugin (to access the online enhanced version of this article).
(PDF)

## References

[1] Hanson PI, Whiteheart SW (2005). AAA+ proteins: Have engine, will work.. Nature Reviews Molecular Cell Biology.

[2] Erzberger JP, Berger JM (2006). Evolutionary relationships and structural mechanisms of AAA plus proteins.. Annual Review of Biophysics and Biomolecular Structure.

[3] White SR, Lauring B (2007). AAA+ ATPases: Achieving diversity of function with conserved machinery.. Traffic.

[4] Snider J, Thibault G, Houry WA (2008). The AAA plus superfamily of functionally diverse proteins.. Genome Biology.

[5] Casari G, De Fusco M, Ciarmatori S, Zeviani M, Mora M (1998). Spastic paraplegia and OXPHOS impairment caused by mutations in paraplegin, a nuclear-encoded mitochondrial metalloprotease.. Cell.

[6] Juhola MK, Shah ZH, Grivell LA, Jacobs HT (2000). The mitochondrial inner membrane AAA metalloprotease family in metazoans.. Febs Letters.

[7] Nolden M, Ehses S, Koppen M, Bernacchia A, Rugarli EI (2005). The m-AAA protease defective in hereditary spastic paraplegia controls ribosome assembly in mitochondria.. Cell.

[8] Salinas S, Proukakis C, Crosby A, Warner TT (2008). Hereditary spastic paraplegia: clinical features and pathogenetic mechanisms.. Lancet Neurology.

[9] Rugarli EI, Langer T (2006). Translating m-AAA protease function in mitochondria to hereditary spastic paraplegia.. Trends in Molecular Medicine.

[10] Kabsch W (1993). Automatic processing of rotation diffraction data from crystals of initially unknown symmetry and cell constants.. Journal of Applied Crystallography.

[11] Vagin A, Teplyakov A (2000). An approach to multi-copy search in molecular replacement.. Acta Crystallographica D Biological Crystallography.

[12] Murshudov GN, Vagin A, Dodson EJ (1997). Refinement of macromolecular structures by the maximum-likelihood method.. Acta Crystallographica D Biological Crystallography.

[13] Painter J, Merritt EA (2006). TLSMD web server for the generation of multi-group TLS models.. Journal of Applied Crystallography.

[14] Emsley P, Cowtan K (2004). Coot: model-building tools for molecular graphics.. Acta Crystallographica D Biological Crystallography.

[15] Lovell SC, Davis IW, Adrendall WB, de Bakker PIW, Word JM (2003). Structure validation by C alpha geometry: phi,psi and C beta deviation.. Proteins-Structure Function and Genetics.

[16] Gouet P, Courcelle E, Stuart DI, Metoz F (1999). ESPript: analysis of multiple sequence alignments in PostScript.. Bioinformatics.

[17] Niwa H, Tsuchiya D, Makyio H, Yoshida M, Morikawa K (2002). Hexameric ring structure of the ATPase domain of the membrane-integrated metalloprotease FtsH from Thermus thermophilus HB8.. Structure.

[18] Briggs LC, Baldwin GS, Miyata N, Kondo HS, Zhang XD (2008). Analysis of nucleotide binding to p97 reveals the properties of a tandem AAA hexameric ATPase.. Journal of Biological Chemistry.

[19] Karata K, Inagawa T, Wilkinson AJ, Tatsuta T, Ogura T (1999). Dissecting the role of a conserved motif (the second region of homology) in the AAA family of ATPases - Site-directed mutagenesis of the ATP-dependent protease FtsH.. Journal of Biological Chemistry.

[20] Suno R, Niwa H, Tsuchiya D, Zhang XD, Yoshida M (2006). Structure of the whole cytosolic region of ATP-dependent protease FtsH.. Molecular Cell.

[21] Zhang XD, Wigley DB (2008). The ‘glutamate switch’ provides a link between ATPase activity and ligand binding in AAA plus proteins.. Nature Structural & Molecular Biology.

[22] Ogura T, Whiteheart SW, Wilkinson AJ (2004). Conserved arginine residues implicated in ATP hydrolysis, nucleotide-sensing, and inter-subunit interactions in AAA and AAA(+) ATPases.. Journal of Structural Biology.

[23] Ammelburg M, Frickey T, Lupas AN (2006). Classification of AAA+ proteins.. Journal of Structural Biology.

[24] Holm L, Kaariainen S, Rosenstrom P, Schenkel A (2008). Searching protein structure databases with DaliLite v.3.. Bioinformatics.

[25] Bieniossek C, Schalch T, Bumann M, Meister M, Meier R (2006). The molecular architecture of the metalloprotease FtsH.. Proceedings of the National Academy of Sciences of the United States of America.

[26] Yamada-Inagawa T, Okuno T, Karata K, Yamanaka K, Ogura T (2003). Conserved pore residues in the AAA protease FtsH are important for proteolysis and its coupling to ATP hydrolysis.. Journal of Biological Chemistry.

[27] Martin A, Baker AT, Sauer AT (2008). Pore loops of the AAA+ ClpX machine grip substrates to drive translocation and unfolding.. Nature Structural & Molecular Biology.

[28] Brugman F, Scheffer H, Wokke JHJ, Nillesen WM, de Visser M (2008). Paraplegin mutations in sporadic adult-onset upper motor neuron syndromes.. Neurology.

[29] Wilkinson PA, Crosby AH, Turner C, Bradley LJ, Ginsberg L (2004). A clinical, genetic and biochemical study of SPG7 mutations in hereditary spastic paraplegia.. Brain.

[30] Elleuch N, Depienne C, Benomar A, Hernandez AMO, Ferrer X (2006). Mutation analysis of the paraplegin gene (SPG7) in patients with hereditary spastic paraplegia.. Neurology.

[31] Arnoldi A, Tonelli A, Crippa F, Villani G, Pacelli C (2008). A clinical, genetic, and biochemical characterization of SPG7 mutations in a large cohort of patients with hereditary spastic paraplegia.. Human Mutation.

[32] Ferreirinha F, Quattrini A, Valsecchi V, Errico A, Ballabio A (2001). Mice lacking paraplegin, a mitochondrial AAA protease involved in hereditary spastic paraplegia, show axonal degeneration and abnormal mitochondria.. American Journal of Human Genetics.

[33] Atorino L, Silvestri L, Koppen M, Cassina L, Ballabio A (2003). Loss of m-AAA protease in mitochondria causes complex I deficiency and increases sensitivity to oxidative stress in hereditary spastic paraplegia.. Journal of Cell Biology.

[34] Koppen M, Metodiev MD, Casari G, Rugarli E, Langer T (2007). Variable and tissue-specific subunit composition of mitochondrial m-AAA protease complexes linked to hereditary spastic paraplegia.. Molecular and Cellular Biology.

